# Global Patterns of Geographic Distribution, Temporal Trends, Host Spectrum, and Molecular Variation of H9N2 Avian Influenza Virus, 1966–2023

**DOI:** 10.3390/v18090977

**Published:** 2026-09-04

**Authors:** Lei Chang, Xiangyan Ren, Lebin Han, Chenlu Xia, Yuzhong Zhao

**Affiliations:** 1 Jining Polytechnic, Jining 272007, China; 2 Shouguang Hualong Town Animal Husbandry and Veterinary Workstation, Weifang 262721, China; 3 State Key Laboratory for Animal Disease Control and Prevention, Lanzhou Veterinary Research Institute, Chinese Academy of Agricultural Sciences, Lanzhou 730046, China; 4 College of Veterinary Medicine, Shandong Agricultural University, Tai’an 271018, China

**Keywords:** H9N2 avian influenza virus, epidemiological characteristics, host, molecular, genetic polymorphism

## Abstract

The H9N2 avian influenza virus (AIV) is currently widespread globally and poses a serious threat to the poultry industry and public health; however, its global epidemiological characteristics and key molecular mutation patterns have not yet been systematically elucidated. Based on the NCBI Influenza Virus Resource Database, this study collected relevant data on the H9N2 AIV from around the world between 1966 and 2023. It conducted a comprehensive analysis of the geographic distribution of 11,933 strains, the temporal distribution of 11,824 strains, the host sources of 11,756 strains, and the genetic polymorphisms at key functional sites. The results showed that 90.14% of H9N2 AIV sequences in the dataset originated from Asia, with China contributing the largest number of sequences. The global sequence availability pattern underwent four phases: sporadic sequence availability, gradual increase, high sequence availability, and subsequent decline, with 2007–2018 representing a period of relatively high availability of publicly accessible sequences. Host analysis indicated that H9N2 AIV sequences were predominantly derived from poultry-related hosts, with chickens being the main host source in the dataset, and available sequences have also been reported from various mammals, including pigs and humans, as well as environmental media. Molecular analysis revealed that receptor-binding-related sites in the HA protein, such as Q226L and I155T, have become dominant variants, while mammalian-adaptive mutations such as PB2 E627K and D701N remain at low frequencies; M2 S31N is widely prevalent, whereas the detection rate of NA drug-resistance-associated mutations is low. This study systematically reveals the global distribution patterns of available H9N2 AIV sequences, host distribution characteristics, and key molecular mutation patterns of the H9N2 AIV, providing a scientific basis for cross-host transmission risk assessment, molecular surveillance, and targeted prevention and control.

## 1. Introduction

The H9N2 subtype of avian influenza virus (AIV) is one of the major subtypes of influenza A virus and was first isolated from turkeys in North America in 1966 [1]. According to their pathogenicity in poultry, AIVs are classified into highly pathogenic avian influenza viruses (HPAIVs) and low pathogenic avian influenza viruses (LPAIVs). Among them, H5- and H7-subtype HPAIVs usually cause high mortality in poultry, whereas the H9N2 subtype is a typical LPAIV that mainly causes mild respiratory symptoms after infection and can also result in reduced egg production and impaired growth performance, leading to considerable economic losses [2,3].

In addition to its widespread circulation in poultry, the H9N2 virus has a strong ability to cross species barriers and can infect a variety of mammals, including pigs, minks, raccoon dogs, and humans [4,5,6]. Although H9N2 virus infections in humans are relatively uncommon compared with its widespread circulation in avian populations, sporadic human cases have been reported in several countries in recent years [7]. These findings suggest that H9N2 AIV has the potential for zoonotic transmission and may pose a public health concern. Notably, the H9N2 virus has dual roles as both a gene donor and a gene recipient. On the one hand, its internal genes can provide the genetic backbone for emerging human-infecting AIVs such as H5N1, H7N9, and H10N3 [8,9]. On the other hand, the H9N2 virus can acquire foreign gene segments from other subtypes through reassortment, thereby promoting continuous viral evolution and enhancing its potential for cross-species transmission [10,11]. Therefore, the H9N2 virus is considered a key hub in the cross-species transmission network of AIVs. Its continued circulation not only poses a long-term threat to the poultry industry but also increases the risk of the emergence and spread of novel influenza viruses.

A comprehensive understanding of the global epidemiological characteristics, host distribution, and molecular variation dynamics of the H9N2 virus is essential for accurately assessing its risk of cross-species transmission and developing effective prevention and control strategies. Although H9N2 viruses have been reported in various regions worldwide, most epidemiological studies have been conducted at national or regional scales, and comprehensive analyses based on long-term global surveillance data remain limited [12,13,14]. At the molecular level, although the biological functions of several key amino acid sites have been extensively investigated [15], systematic analyses of the genetic polymorphism and epidemiological characteristics of key functional sites among globally circulating strains are still lacking. In particular, comprehensive evaluations of the prevalence and potential risks of different mutation sites remain insufficient. These limitations have hindered a comprehensive understanding of the global epidemiology of H9N2 virus and its risk of cross-species transmission. Furthermore, despite the use of vaccines and antiviral drugs, controlling H9N2 AIV remains challenging due to its continuous evolution [16,17]. Changes in viral antigens may reduce vaccine protection, while the emergence of drug-resistant strains may limit treatment options. Therefore, continuous monitoring of viral evolution is important for improving H9N2 prevention and control strategies.

In this study, based on the influenza virus resource database of the National Center for Biotechnology Information (NCBI), we systematically collected strain information and gene sequence data of global H9N2 subtype AIVs from 1966 to 2023 and performed a comprehensive analysis from three aspects: spatiotemporal distribution, host distribution, and molecular characteristics. At the epidemiological level, global geographic distribution data and annual time-series data were constructed based on the geographic information of 11,933 strains and the isolation dates of 11,824 strains to systematically characterize the global distribution pattern and long-term epidemiological trends of the H9N2 virus. At the host level, host and environmental source information from 11,756 H9N2 AIV strains was analyzed to characterize the distribution patterns of the virus among avian hosts, mammalian hosts, and environmental sources, and to evaluate its host range, environmental occurrence, and potential zoonotic risk. At the molecular level, we investigated important functional sites in the viral polymerase complex (PB2, PB1, and PA), nucleoprotein (NP), matrix proteins (M1 and M2), hemagglutinin (HA), and neuraminidase (NA), and determined the composition and frequency of different amino acid residues among globally circulating strains. In addition, the prevalence and potential risks of key mutation sites were systematically evaluated.

This study aims to improve the epidemiological and molecular mutation data of the global H9N2 subtype AIV and to provide comprehensive data support and a theoretical basis for establishing region-specific prevention and control strategies, integrated host surveillance systems, and molecular target-based early warning systems. It is expected to contribute to the scientific control of H9N2 avian influenza and the early warning of its cross-species transmission risk worldwide.

## 2. Materials and Methods

### 2.1. Data Source and Virus Strain Collection

The epidemiological information and genome sequence data of H9N2 AIVs used in this study were obtained from the NCBI Influenza Virus Resource database (https://www.ncbi.nlm.nih.gov/genomes/FLU/Database/nph-select.cgi?go=database (accessed on 3 January 2026)). H9N2 AIV records collected from 1966 to 2023 were downloaded from the database. Virus records clearly identified as H9N2 and containing relevant information, including sampling year, geographic origin, and host information, were included. Records with missing important information or unclear subtype classification were excluded. For epidemiological analysis, virus strains were used as the basic units. Because geographic information, sampling dates, and host information were not available for all strains, the numbers of strains used for geographical distribution analysis, temporal distribution analysis, and host source analysis were different. For genetic sequence analysis, eight gene segments of H9N2 AIVs, including HA, NA, PB2, PB1, PA, NP, M, and NS, were selected. Different gene segments from the same virus strain were analyzed separately. The number of sequences analyzed for each gene segment varied because the numbers of available sequences and target amino acid sites differed among gene segments. Duplicate records were checked based on strain name, sampling date, host source, and geographic origin, and duplicate entries were removed. Each virus strain was counted only once in the epidemiological analysis.

### 2.2. Global Spatiotemporal Distribution Analysis

Based on the geographic information (*n* = 11,933) and temporal information (*n* = 11,824) of the included H9N2 AIV strain, global spatial distribution and temporal distribution datasets were established, respectively. Geographic distribution was analyzed at the country level by calculating the numbers of virus strains detected in each continent and country/region to characterize the global spatial distribution pattern and regional clustering characteristics. Temporal distribution was analyzed on an annual basis by calculating the number of virus strains identified each year from 1966 to 2023. Based on annual changes in strain numbers, together with changes in sequence availability, different temporal stages of sequence availability were defined to describe the long-term changes in the publicly available H9N2 AIV sequence dataset.

### 2.3. Host and Environmental Source Analysis

According to the host type and sample source annotated for each virus strain, a total of 11,756 strains were classified into three categories: avian hosts, mammalian hosts, and environmental sources. Avian hosts were further divided into domestic poultry (including chickens, ducks, pigeons, quails, and turkeys) and wild birds to analyze distribution differences among avian species. Mammalian hosts included domestic mammals, wild mammals, and humans to evaluate the range of cross-species infection. Environmental strains included general environmental strains, cage strains, air strains, and fecal strains to analyze the environmental occurrence and transmission characteristics of the virus, thereby systematically characterizing the host spectrum and environmental transmission patterns of H9N2 virus.

### 2.4. Sequence Alignment and Analysis of Key Amino Acid Sites

Nucleotide sequences of individual gene segments of H9N2 AIV, including HA, NA, PB2, PB1, PA, NP, M1, and M2, were downloaded from the NCBI Influenza Virus Resource database. Multiple sequence alignment was performed using Clustal Omega (v1.2.4) (http://www.clustal.org/omega/ (accessed on 11 February 2026)), and sequence organization and format conversion were conducted using MEGA (v7.0) [18]. Key amino acid substitutions in H9N2 AIV proteins, including PB2, PB1, PA, NP, M1, M2, HA, and NA, were analyzed based on previously reported functional sites associated with viral replication, receptor binding, pathogenicity, immune escape, and antiviral resistance [19,20,21,22,23,24,25,26,27,28,29,30,31,32,33,34,35,36,37,38]. By systematically comparing the occurrence of mutations at these key sites, the molecular mutation characteristics and potential genetic variation risks of H9N2 AIV were evaluated.

### 2.5. Statistical Analysis

All data were organized, summarized, and statistically analyzed using Excel 2021. The distribution of virus strains across different geographic regions, time periods, and host categories, as well as the occurrence of wild-type and mutant amino acids at key sites, were calculated. Descriptive statistical methods were used to analyze the global spatiotemporal distribution, host source characteristics, and molecular variation patterns of H9N2 AIV. The risks of viral transmission and evolution were further evaluated in combination with the epidemiological dynamics and molecular variation characteristics of the virus. All analyses were based on publicly available database records, ensuring the objectivity and reproducibility of the analytical process.

## 3. Results

### 3.1. Geographical Distribution Characteristics

**Figure 1:**
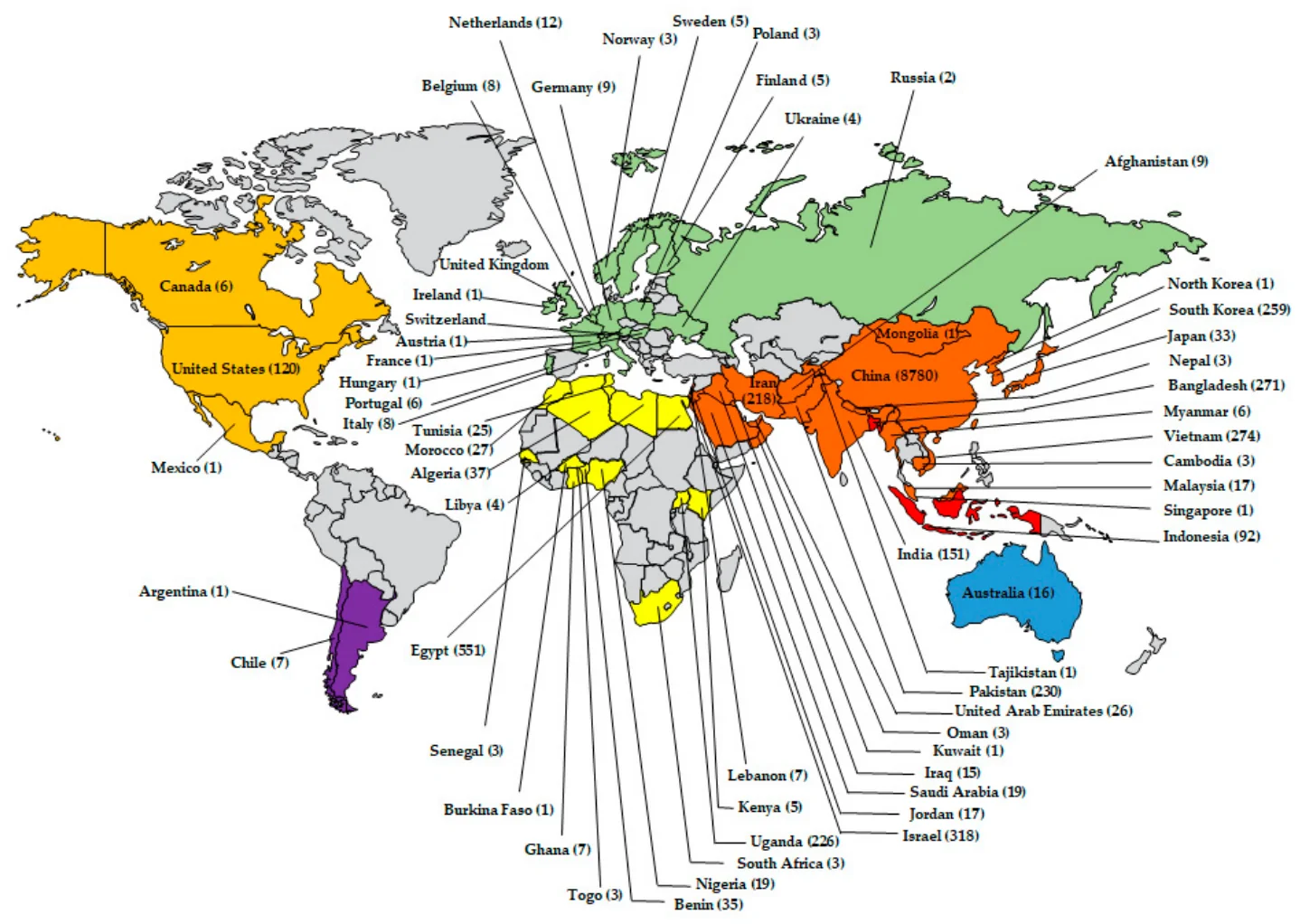
Global geographical distribution of H9N2 AIV strains. The map illustrates the global distribution of 11,933 H9N2 AIV strains collected from 63 countries across six continents. Different colors represent different continents, and the numbers in parentheses indicate the number of H9N2 AIV strains reported in each country.

A total of 11,933 H9N2 AIV strains were collected in this study, covering 63 countries across six continents. Based on these data, the global geographical distribution characteristics were analyzed (Figure 1). The results showed that H9N2 AIV exhibited an uneven spatial distribution worldwide, with a marked pattern of regional clustering, predominantly in Asia. A total of 26 countries in Asia were reported to be positive for the virus, with 10,756 strains in total, accounting for 90.14% of the global total, indicating that Asia contributed the largest proportion of available H9N2 AIV sequences in this dataset. China had the highest number of strains (8780), representing the largest source of sequences included in this analysis. This was followed by Israel (318), Vietnam (274), Bangladesh (271), South Korea (259), Pakistan (230), Iran (218), India (151), and Indonesia (92), while other Asian countries reported relatively low numbers. In Europe, H9N2 strains were detected in 17 countries, with a total of 80 strains. The Netherlands (12), Germany (9), Belgium (8), Italy (8), and Switzerland (8) reported relatively higher numbers, while other countries had sporadic and limited detections. In Africa, a total of 946 strains were reported, with most strains found in Egypt (551 strains) and Uganda (226 strains), which contributed the majority of African strains in this dataset, while other countries reported fewer strains. In North America, a total of 127 strains were reported from three countries, with the United States being the main source (120). In South America, only Argentina and Chile reported H9N2 strains, with a total of 8 cases. In Oceania, only Australia reported the virus, with 16 strains. Overall, the H9N2 AIV sequences analyzed in this study showed a highly uneven global geographical distribution, with Asia accounting for the largest proportion of available sequences, while other continents showed relatively limited sequence representation.

### 3.2. Temporal Distribution Characteristics

**Figure 2:**
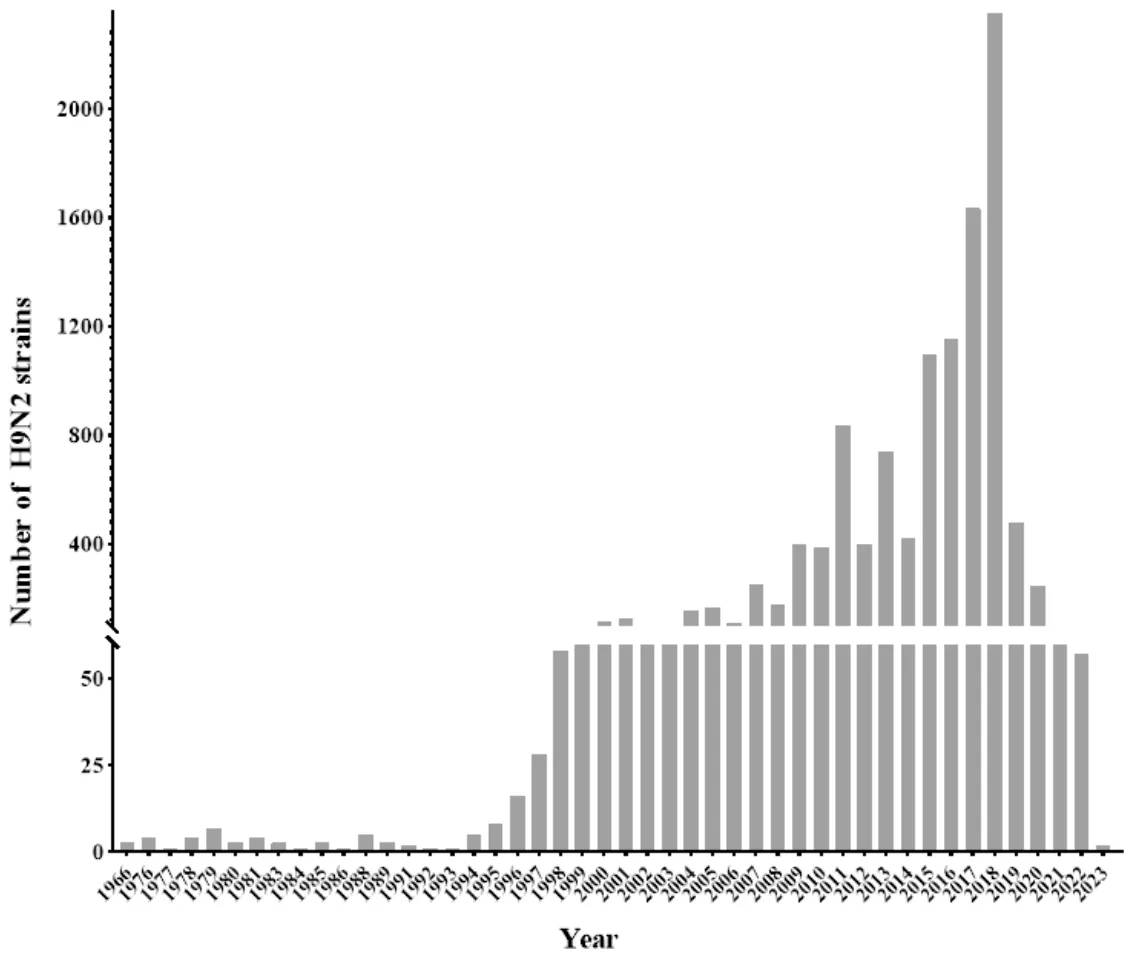
Temporal distribution of the number of H9N2 AIV strains in different years. The figure shows the annual variation trend of H9N2 AIV strains from 1966 to 2023.

Based on global H9N2 AIV sequence data available in the NCBI Influenza Virus Resource Database from 1966 to 2023, the temporal distribution characteristics were analyzed (Figure 2). The results showed that the annual number of available H9N2 AIV sequences exhibited clear stage-specific temporal patterns over time, generally following a progression from sporadic sequence availability, gradual increase, high sequence availability, and subsequent decline. From 1966 to 1995, the available sequence data showed a sporadic detection pattern. During this period, the number of available sequences was limited, with annual sequence numbers mainly ranging from 1 to 8, and no sustained increase in sequence availability was observed. From 1996 to 2006, the number of available sequences gradually increased. After 1996, sequence numbers increased from 16 in 1996 to 72 in 1999 and remained at 96–168 sequences per year during 2000–2006, representing a significant increase in the availability of H9N2 AIV sequences compared with the previous period. From 2007 to 2018, a period of high sequence availability was observed. In 2007, the number of available sequences increased to 251, followed by fluctuating changes, reaching 838 sequences in 2011 and decreasing to 400 sequences in 2012. After 2013, the number of available sequences further increased, with rapid growth during 2015–2018 and a peak of 2350 sequences reached in 2018, representing the highest annual number of sequences during the study period. From 2019 to 2023, a decline in available sequence numbers was observed. After the 2018 peak, annual sequence numbers continuously decreased, declining from 478 sequences in 2019 to only 2 sequences in 2023, indicating a substantial reduction in publicly available H9N2 AIV sequence data during this period. In summary, from 1966 to 2023, H9N2 AIV sequences in the analyzed dataset exhibited clear stage-specific temporal variation, which can be divided into four phases: sporadic sequence availability, gradual increase, high sequence availability, and subsequent decline.

### 3.3. Host and Environmental Source Characteristics

A total of 11,756 strains were analyzed to characterize the host distribution and environmental sources of H9N2 AIV (Table 1). Strains were classified into three categories: avian hosts, mammalian hosts, and environmental sources, representing different ecological origins and transmission media. Avian hosts were the predominant source, accounting for 11,356 strains (96.60%). Chickens represented the largest proportion (9592 strains), serving as the primary host source. Ducks, pigeons, quails, and turkeys also accounted for a proportion of strains. In addition, multiple wild bird species were included, indicating that H9N2 virus is widely distributed across different avian ecological niches and has a broad host range. Mammalian hosts accounted for 114 strains (0.96%), including domestic mammal-, wild mammal-, and human-derived strains. Pig-derived strains were the most frequent (51), representing the primary mammalian host. Human-derived strains ranked second (29), suggesting a certain zoonotic transmission risk. Other sources included mink, raccoon dog, Asian badger, bats, and palm civets, as well as a small number of dogs, horses, and ferrets, indicating that the virus can infect multiple mammalian species. Environmental sources accounted for 286 strains (2.43%), including environment samples, cage swabs, environmental air samples, fecal samples, pigeon feces, and wild bird feces. Among these, 260 strains were annotated as “Environment” in the original database, but detailed sampling information was unavailable. Therefore, these strains were classified as environmental sources based on the available metadata. These findings indicate that H9N2 AIV can not only persist in avian hosts but can also be detected in diverse environmental matrices, suggesting potential environmental contamination and circulation.

### 3.4. Distribution of Functional Amino Acid Variants in H9N2 AIV

To characterize the molecular mutation features of H9N2 AIV strains, representative amino acid substitutions associated with viral replication, host adaptation, receptor binding, antigenic changes, and antiviral drug resistance were analyzed based on a global sequence dataset. The distribution of key amino acid substitutions showed differences among viral proteins. Some substitutions were detected at low frequencies in circulating viral populations, whereas others gradually became common variants during long-term transmission. The representative amino acid substitutions, their reported biological functions, and amino acid distribution patterns are summarized in Table 2. The complete distribution profiles of all analyzed amino acid substitutions are provided in Supplementary Table S1.

Among polymerase complex-related proteins, most amino acid substitutions associated with mammalian adaptation and increased viral replication showed low frequencies. In the PB2 protein, mammalian adaptation-related substitutions, including N253, L404, K591, K627, N701, and R714, were rarely detected. In contrast, the I292V substitution was relatively common, with the V variant detected in 2509 sequences and the I variant detected in 1733 sequences. In the PB1 protein, the K577 variant remained dominant, whereas the E variant was detected only three times. Similarly, several important substitutions in the PA protein, including T97I, I545V, and S594G, were rarely detected, while the K356R site showed a relatively similar distribution, with K and R variants detected in 2720 and 2142 sequences, respectively. The E434K substitution in the NP protein was extremely rare, with only one sequence carrying the K variant. These results suggest that although some polymerase-related mutations have been shown experimentally to contribute to mammalian adaptation, they have not become common among currently circulating H9N2 AIV strains (Table 2).

In the matrix proteins, M1-T37A and M2-S31N substitutions showed clear differences in variant distribution. At position 37 of the M1 protein, the A variant was detected in 3173 sequences, which was higher than the number of sequences carrying the original T variant (1677 sequences). For the M2 protein, the N variant at position 31 was detected in 3355 sequences, while the S variant was detected in 1688 sequences, indicating that the S31N substitution associated with adamantane resistance has become a common variant among currently circulating H9N2 AIV populations (Table 2).

The HA protein showed a high level of amino acid variation, especially at sites related to receptor binding, host adaptation, and antigenic properties. Different amino acid variants showed different distribution patterns at host adaptation- and receptor binding-related sites. At the T137I site, the T variant was detected in 1495 sequences, whereas the I variant was detected in only one sequence. At the I155T site, the T variant was the major variant, detected in 11,592 sequences, while the I variant was detected in only seven sequences. At the N166D site, N and D variants were detected in 8658 and 2120 sequences, respectively. At the A190V site, the A variant (4644 sequences) was more frequent than the V variant (1178 sequences). At the Q226L site, the L variant was detected in 10,562 sequences, whereas the Q variant was detected in 1108 sequences. At the R246K site, K and R variants were detected in 6408 and 5279 sequences, respectively. Other receptor-binding-related sites also showed different levels of amino acid variation. At the Q227M site, Q and M variants were detected in 3099 and 6710 sequences, respectively. The D145G/N site contained three variants, including D, G, and N, which were detected in 6116, 4352, and 1056 sequences, respectively. At the S119R site, S and R variants were detected in 3891 and 7241 sequences, respectively (Table 2).

Further analysis of HA sites related to binding to human α2,6-linked sialic acid receptors showed that several sites contained frequently detected variant amino acids. At the A160D/N site, A, D, and N variants were detected in 4109, 1312, and 3005 sequences, respectively. At the Q156R site, Q and R variants were detected in 6756 and 4256 sequences, respectively. At the T205A site, the number of A variants was higher than that of T variants (8853 versus 2827 sequences). At the Q226L site, the L variant was detected in 10,562 sequences, whereas the Q variant was detected in 1108 sequences. At the V245I site, the I variant was detected in 8362 sequences, while the V variant was detected in 3350 sequences. At the V216L site, L and V variants were detected in 10,569 and 915 sequences, respectively. At the D208E site, the E variant was detected in 8082 sequences, whereas the D variant was detected in 1855 sequences. At the T212I site, T and I variants showed similar frequencies, with 5388 and 5665 sequences detected, respectively. At the R172Q and S175N sites, Q and N variants were detected in 7857 and 9099 sequences, respectively (Table 2).

At antigenic sites, different amino acid substitutions also showed different distribution patterns. At the D127S site, D and S variants were detected in 4321 and 5132 sequences, respectively. At the G135D site, G and D variants were detected in 4352 and 6116 sequences, respectively. At the N145T site, the T variant was detected in 11,592 sequences, whereas the N variant was detected in only 50 sequences. At the R146Q site, R and Q variants were detected in 4256 and 6756 sequences, respectively. At the D179T site, D and T variants were detected in 2454 and 8709 sequences, respectively. At the R182T site, R and T variants were detected in 4060 and 6938 sequences, respectively. At the T183N site, T and N variants were detected in 114 and 8314 sequences, respectively (Table 2).

For the NA protein, amino acid substitutions related to antiviral drug resistance remained uncommon. The E119D substitution was detected only three times, and the R292K substitution was not found in the analyzed dataset, suggesting that these resistance-related substitutions have not yet become common among circulating H9N2 AIV populations (Table 2).

Overall, amino acid distribution analysis showed that most polymerase complex mutations related to mammalian adaptation remain at low frequencies, whereas multiple HA substitutions related to receptor binding and antigenic changes are already widely present in current H9N2 AIV strains. These findings suggest that H9N2 AIV has continuously accumulated important mutations affecting host adaptation and antigenic properties during long-term transmission. Continuous molecular surveillance is therefore important for monitoring viral evolution and evaluating potential public health risks.

## 4. Discussion

This study systematically analyzed the spatiotemporal distribution characteristics, host spectrum structure, and molecular mutation patterns of H9N2 AIV based on global sequence and epidemiological data from 1966 to 2023. The results demonstrated that H9N2 AIV sequences included in this dataset showed a highly uneven geographical distribution, with Asia contributing the largest proportion of available sequences. This pattern is highly consistent with previous reports describing Asia as an important region where H9N2 AIV has been frequently detected and reported [39]. This distribution pattern may be driven by the combined effects of high-density poultry farming systems, the long-term presence of live poultry markets, and cross-regional poultry trade, thereby maintaining sustained local transmission cycles [40,41]. It has been proposed that H9N2 AIV circulating in Asian poultry populations is one of the most likely candidate viruses for causing a future human influenza pandemic [42]. This feature highlights the strong geographical linkage of Asia within the AIV ecosystem and suggests a close association with the emergence of cross-species transmission risk.

Temporal analysis revealed substantial year-to-year variation in the availability of H9N2 AIV sequences in public databases, characterized by four main stages: sporadic detection, gradual increase, high sequence availability, and subsequent decline. Sequence availability increased markedly from 2007 onward and remained relatively high through 2018, when the largest number of sequences (2350) was recorded. These sequences represented approximately 19.9% of the 11,824 strains included in the temporal analysis. In contrast, only two sequences were available for 2023, corresponding to a 99.9% decrease compared with the 2018 peak. However, these temporal changes should be interpreted cautiously because the dataset was derived from publicly available records rather than a standardized global surveillance system. Previous studies have shown that avian influenza surveillance outcomes can be influenced by sampling design, sampling intensity, surveillance coverage, and the timing and geographic location of sampling [43,44]. Differences in surveillance practices among regions may therefore contribute to substantial variation in the number and distribution of viruses represented in public databases. Accordingly, the marked decline in available H9N2 sequences after 2018 should not be interpreted directly as evidence of reduced H9N2 circulation. Rather, the temporal pattern observed here primarily reflects changes in the availability of H9N2 genomic sequences in public databases, which may be influenced by variation in surveillance and sequencing efforts. Continued standardized surveillance integrating epidemiological investigations, virus isolation, and genomic sequencing will be essential for improving our understanding of the long-term circulation and evolutionary dynamics of H9N2 viruses.

Host analysis showed that available H9N2 AIV sequences were mainly obtained from poultry hosts, with chickens being the most common host in this dataset. Ducks, pigeons, and quails were also identified as important avian hosts in different ecological settings. These findings indicate that H9N2 viruses have been detected in a wide range of avian hosts and may have the ability to adapt to different poultry species. Previous studies have demonstrated that H9N2 viruses are not only widely distributed among poultry populations but can also be detected in wild birds, environmental samples, and certain mammalian hosts, highlighting their broad ecological distribution and potential adaptability across diverse host and environmental niches [45,46,47]. Although sequences from mammalian hosts accounted for a relatively small proportion, H9N2 viruses have been reported in pigs and humans, indicating that cross-species infection events have occurred [45]. Pigs are considered potential intermediate hosts because they can be infected by both avian and mammalian influenza viruses, which may facilitate viral reassortment and contribute to the emergence of viruses with zoonotic potential [48]. A study reported a Eurasian avian-like H1N1 swine influenza virus in which the PB1 and M gene segments were derived from H9N2 viruses, providing direct evidence that H9N2 viruses can contribute to natural reassortment with swine influenza viruses and suggesting that H9N2 viruses may play an important role in influenza virus reassortment and the emergence of novel viruses [49]. In addition, the continuous reports of human infections with H9N2 viruses further highlight their potential zoonotic risk; however, compared with HPAIVs, human infections with H9N2 viruses are currently characterized mainly by sporadic cases, and most reported cases exhibit mild clinical symptoms, with no evidence of sustained human-to-human transmission [50]. Therefore, the public health impact of H9N2 viruses requires further evaluation based on infection frequency, clinical severity, and transmission potential. Furthermore, the detection of H9N2 viral genetic material in environmental samples, including farm environments, feces, and air, suggests that environmental contamination may contribute to viral spread. However, the detection of viral nucleic acids alone does not confirm that the virus remains infectious or can be transmitted, and further studies involving virus isolation and experimental validation are needed to clarify the role of environmental sources in H9N2 transmission.

This study characterized the polymorphism and population distribution of key amino acid sites in available H9N2 AIV sequences and identified several variants with potential functional significance. Multiple low-frequency amino acid substitutions were detected in the PB2, PB1, PA, and NP proteins, some of which have been reported to be associated with viral polymerase activity or mammalian host adaptation. For example, PB2-E627K, PB2-D701N, PB2-Q591K, PB1-K577E, and PA-T97I have been reported to potentially enhance influenza virus polymerase activity or viral replication [22,23,24,25,26]. Although these variants occurred at relatively low frequencies in the available H9N2 sequences analyzed in this study, their potential biological effects warrant further attention, and these sites may serve as candidates for future functional studies. HA is an important viral surface protein that plays a central role in receptor binding and antigenicity. In the present study, variants such as I155T [31], Q226L [34], and V245I [36] occurred at relatively high frequencies in H9N2 virus sequences, suggesting that these sites may be under ongoing selection pressure. Previous studies have shown that some HA amino acid substitutions can alter receptor-binding properties and host adaptation, while long-term immune pressure in poultry populations may drive antigenic drift in H9N2 viruses, resulting in antigenic differences between circulating strains and vaccine strains and potentially affecting vaccine protection [51]. Therefore, continued surveillance of mutations at HA receptor-binding sites and antigenic sites is important for assessing antigenic evolution, vaccine matching, and future epidemiological trends of H9N2 viruses. Notably, combinations of functionally relevant mutations may have more complex biological effects than individual substitutions. HA-Q226L and I155T have been associated with changes in receptor-binding properties and host adaptation, whereas the relatively low-frequency PB2-E627K and D701N substitutions have been associated with increased polymerase activity and mammalian host adaptation. Although mutation co-occurrence was not further analyzed in the present study, the simultaneous acquisition and subsequent fixation of HA receptor-binding mutations and PB2 mutations associated with mammalian adaptation in naturally circulating H9N2 viruses could theoretically affect both receptor usage and viral replication capacity during cross-species adaptation, and therefore warrants continued monitoring. In particular, if such functionally relevant mutations continue to occur or increase in frequency in natural isolates, their genetic backgrounds and epidemiological trends should be closely monitored. However, this potential risk is inferred from the known functions of these sites reported in previous studies and does not by itself indicate increased human-to-human transmissibility or pandemic potential. Host adaptation and transmission are determined by multiple viral genes and their overall genetic background; therefore, the actual biological effects of these mutation combinations require further evaluation using reverse genetics, polymerase activity assays, receptor-binding assays, and viral replication experiments. In addition, M2-S31N was detected at a high frequency among H9N2 viruses worldwide, suggesting that resistance to adamantane drugs is already widespread, consistent with the long-term emergence and spread of resistance to M2 ion channel inhibitors among influenza A viruses [52]. In contrast, mutations associated with neuraminidase inhibitor resistance occurred at relatively low frequencies in the present dataset and had not become predominant variants. Because the emergence and spread of antiviral resistance mutations are influenced by genetic background, drug selection pressure, and opportunities for viral transmission, continued surveillance of these sites remains necessary. It should be emphasized that sequence frequency mainly reflects the distribution of a specific variant among currently available viral sequences and cannot, by itself, indicate its biological effect or epidemiological advantage. Therefore, the findings of this study, based on sequence frequencies and functional information from previous studies, are mainly intended to describe the molecular variation patterns of H9N2 viruses and identify potentially important sites. Future studies should integrate phylogenetic background, longitudinal surveillance data, and the overall genetic background of the virus, together with functional experiments, to further clarify the relationship between molecular evolution and phenotypic changes in H9N2 viruses.

Based on the epidemiological and molecular characteristics revealed in this study, traditional homogeneous and experience-based control strategies are no longer sufficient to address the current epidemic situation of H9N2. Given the markedly uneven global distribution of available viral sequences, implementing region-specific hierarchical control is essential to improve control efficiency. A three-tier system—strict control in regions with frequent H9N2 detection, precise control in local endemic regions, and prevention of introduction in sporadic regions—can better match regional risk profiles and avoid inefficient allocation of control resources. Meanwhile, relying on the full host transmission chain of the virus, an integrated control system covering poultry, wild birds, mammals, and the environment can be established to interrupt viral transmission cycles at the source. In addition, a dynamic surveillance system based on molecular variation characteristics, combined with vaccine updates and optimization of clinical antiviral use, can facilitate a transition from passive response to active early warning, forming a closed-loop system of “surveillance–early warning–control–optimization”.

This study has several limitations. First, the analysis was based on available H9N2 AIV sequences from public databases rather than a standardized global active surveillance system. Therefore, the observed patterns of spatial and temporal distribution, host composition, and epidemiological trends may be affected by differences in global surveillance intensity, sequencing capacity, and data-sharing practices. Due to uneven sampling strategies and data availability among regions, the current dataset inevitably contains geographic, temporal, and host sampling biases, which may limit its ability to fully represent the actual global epidemiological characteristics of H9N2 AIV [43,44,53]. For example, the low detection rates of H9N2 AIV in regions with limited available data should not be simply interpreted as the absence of viruses, but may instead reflect insufficient surveillance coverage. Therefore, the regional distribution patterns and circulation dynamics identified in this study should be interpreted as findings based on currently available data and should be further evaluated using more systematic and balanced global surveillance datasets.

## 5. Conclusions

In this study, we analyzed the global distribution, host origins, and key molecular variations in H9N2 AIVs based on epidemiological data and genome sequences collected from 1966 to 2023. The results showed that H9N2 AIV sequences exhibited different distribution patterns among regions, with Asia representing the region with the largest proportion of available virus sequences. In addition, the detection of H9N2 viruses in different bird species, mammals, and environmental samples suggests that H9N2 AIVs have a broad host distribution. Molecular analysis showed that H9N2 AIVs have continued to undergo genetic changes during long-term circulation. Although mutations related to mammalian adaptation remain at low frequencies in current strains, some mutations associated with HA receptor binding, antigenic properties, and M2 antiviral resistance are commonly found in circulating viruses. These results indicate that continuous monitoring of key molecular sites is needed to better understand the genetic changes in H9N2 AIVs.

Overall, this study summarizes the global distribution patterns of available H9N2 AIV sequences and their molecular variation characteristics and provides useful data for future virus surveillance and risk assessment. Continued monitoring of epidemiological information, host origins, and molecular characteristics will improve the understanding of H9N2 AIV evolution and provide support for the development of appropriate prevention and control measures.

## Figures and Tables

**Table 1:** Host and environmental origins of H9N2 AIV strains.

Category	Total Number of Strains	Detailed Sources and Numbers
Avian hosts	11,356	Accipiter gentilis schvedowi (1); African stonechat (1); American black duck (2); American oystercatcher (1); American wigeon (1); Mallard (84); Goose (57); Grey goose (1); Cackling goose (1); Emperor goose (1); Pink-footed goose (1); White-fronted goose (10); Snow goose (2); Bean goose (7); Demoiselle crane (2); Little owl (1); Chestnut teal (1); Bewick’s swan (2); Black swan (1); Chestnut teal (3); Chicken (9592); Red junglefowl (8); Guinea fowl (2); Chinese hwamei (1); Chinese francolin (1); House crow (1); Mute swan (Cygnus olor) (3); Duck (766); Eurasian teal (27); Muscovy duck (27); Spot-billed duck (7); Mandarin duck (1); Eurasian wigeon (2); Falcated duck (2); Common kestrel (2); Flamingo (2); Quail (171); Northern pintail (10); Northern shoveler (8); Sparrow (13); Peacock (4); Pheasant (48); Pigeon (273); Ruddy turnstone (19); Striated heron (1); Baikal teal (1); Black-winged stilt (1); Black-billed magpie (3); Brambling (1); Cattle egret (3); Coot (2); Carrion crow (1); Gadwall duck (3); Garganey (1); Grey-headed gull (1); Laughing gull (1); Great bustard (1); Green peafowl (2); Grey teal (1); Houbara bustard (1); Common myna (1); Ostrich (10); Parakeet (2); Partridge (37); Rosy-billed pochard (1); Ruddy shelduck (1); Sanderling (2); Silver pheasant (1); Stone curlew (2); Thick-billed murre (2); Turkey (98); White-bellied bustard (5)
Mammalian hosts	114	Asian badger (2); Bat (2); Mink (15); Swine (51); Dog (1); Human (29); Horse (2); Ferret (1); Red fox (1); Masked palm civet (2); Pika (1); Raccoon dog (7)
Environmental strains	286	Environment (260); Cage swab (5); Environmental air (4); Feces (10); Pigeon feces (1); Wild bird feces (6)

Note: Environmental sources include H9N2 AIV strains obtained from non-host environmental samples. The category “Environment” follows the original database annotation, where detailed sampling information was unavailable.

**Table 2:** Representative biologically important amino acid substitutions and their distributions in circulating H9N2 AIV strains.

Protein	Mutation Site	Biological Significance	Amino Acid Distribution	High-Frequency Variants
PB2	D253N	Enhances polymerase activity and replication capacity in human cells, without increasing pathogenicity in mice [19]	D (4417); N (5)	No
	F404L	Enhances polymerase activity and increases pathogenicity of multiple influenza subtypes in mice [20]	F (4738); L (1)	No
	Q591K	Enhances replication in human bronchial epithelial cells; weaker than E627K; partially compensates for E627K loss with genetic background dependency [21]	Q (4661); K (51)	No
	E627K	Increase polymerase activity, in vitro replication, and mouse pathogenicity [22]	E (4503); K (26)	No
	D701N	Increase polymerase activity, in vitro replication, and mouse pathogenicity [22]	D (4753); N (16)	No
	S714R	Increase polymerase activity, in vitro replication, and mouse pathogenicity [22]	S (4610); R (0)	No
	I292V	Increases polymerase activity, suppresses IFN-β response, and enhances replication and pathogenicity in mice [23]	I (1733); V (2509)	Yes
PB1	K577E	Enhances polymerase activity, in-creases replication in mouse nasal turbinates, and causes 100% mortality in infected mice [24]	K (4411); E (3)	No
PA	T97I	Enhance polymerase activity in mammalian cells and promote viral replication in vitro and in vivo [25]	T (4355); I (3)	No
	I545V	Enhance polymerase activity in mammalian cells and promote viral replication in vitro and in vivo [25]	I (4532); V (267)	No
	S594G	Enhance polymerase activity in mammalian cells and promote viral replication in vitro and in vivo [25]	S (4760); G (5)	No
	K356R	Enhances polymerase activity and increases mouse pathogenicity [26]	K (2720); R (2142)	No
NP	E434K	Enhances polymerase activity [27]	E (4554); K (1)	No
M1	T37A	Increases viral replication and virulence in human cells and mice [28]	T (1677); A (3173)	Yes
M2	S31N	Alters M2 ion channel conformation and confers resistance to adamantane antivirals [29]	S (1688); N (3355)	Yes
HA	T137I	Enhances viral replication and guinea pig transmission, and increases affinity for human α2,6-linked sialic acid receptors [30]	T (1495); I (1)	No
	I155T	Confers preferential binding to human α2,6-linked sialic acid receptors [31]	I (7); T (11,592)	Yes
	N166D	Alters antigenicity, reduces mouse pathogenicity, and weakens hemagglutination inhibition antibody response in chickens [32]	N (8658); D (2120)	No
	A190V	Enhances binding affinity to mouse and human lung tissues and A549 cells without changing specificity for α2,6-linked sialic acid receptors [33]	A (4644); V (1178)	No
	Q226L	Alters cell tropism, favoring infection of non-ciliated human airway cells and supporting respiratory droplet transmission in ferrets [34]	Q (1108); L (10,562)	Yes
	R246K	Enhances replication in human lung epithelial cells, increases early oropharyngeal shedding in chickens, and increases mouse pathogenicity [35]	R (5279); K (6408)	Yes
	Q227M	Enhance binding to both avian α2,3 and human α2,6-linked sialic acid receptors [36]	Q (3099); M (6710)	Yes
	D145G/N	Enhance binding to both avian α2,3 and human α2,6-linked sialic acid receptors [36]	D (6116); G (4352); N (1056)	No
	S119R	Enhance binding to both avian α2,3 and human α2,6-linked sialic acid receptors [36]	S (3891); R (7241)	Yes
	R246K	Enhance binding to both avian α2,3 and human α2,6-linked sialic acid receptors [36]	R (5279); K (6408)	Yes
	A160D/N	Enhance binding to human α2,6-linked sialic acid receptors [36]	A (4109); D (1312); N (3005)	No
	Q156R	Enhance binding to human α2,6-linked sialic acid receptors [36]	Q (6756); R (4256)	No
	T205A	Enhance binding to human α2,6-linked sialic acid receptors [36]	T (2827); A (8853)	Yes
	Q226L	Enhance binding to human α2,6-linked sialic acid receptors [36]	Q (1108); L (10,562)	Yes
	V245I	Enhance binding to human α2,6-linked sialic acid receptors [36]	V (3350); I (8362)	Yes
	V216L	Enhance binding to human α2,6-linked sialic acid receptors [36]	V (915); L (10,569)	Yes
	D208E	Enhance binding to human α2,6-linked sialic acid receptors [36]	D (1855); E (8082)	Yes
	T212I	Enhance binding to human α2,6-linked sialic acid receptors [36]	T (5388); I (5665)	Yes
	R172Q	Enhance binding to human α2,6-linked sialic acid receptors [36]	R (3137); Q (7857)	Yes
	S175N	Enhance binding to human α2,6-linked sialic acid receptors [36]	S (681); N (9099)	Yes
	D127S	Alter antigenic properties of H9N2 strains [37]	D (4321); S (5132)	No
	G135D	Alter antigenic properties of H9N2 strains [37]	G (4352); D (6116)	Yes
	N145T	Alter antigenic properties of H9N2 strains [37]	N (50); T (11,592)	Yes
	R146Q	Alter antigenic properties of H9N2 strains [37]	R (4256); Q (6756)	Yes
	D179T	Alter antigenic properties of H9N2 strains [37]	D (2454); T (8709)	Yes
	R182T	Alter antigenic properties of H9N2 strains [37]	R (4060); T (6938)	Yes
	T183N	Alter antigenic properties of H9N2 strains [37]	T (114); N (8314)	Yes
NA	E119D	Confers resistance to zanamivir [38]	E (5805); D (3)	No
	R292K	Confers resistance to oseltamivir [38]	R (5854); K (0)	No

Note: PB2, polymerase basic protein 2; PB1, polymerase basic protein 1; PA, polymerase acidic protein; NP, nucleoprotein; M1, matrix protein 1; M2, matrix protein 2; HA, hemagglutinin; NA, neuraminidase. High-frequency variants were defined according to their prevalence and predominance among the analyzed sequences.

## Data Availability

The data were obtained from the following publicly accessible resources in the public domain: NCBI Influenza Virus Resource, URL: https://www.ncbi.nlm.nih.gov/genomes/FLU/Database/nph-select.cgi?go=database (accessed on 3 January 2026).

## Supplementary Materials

The following supporting information can be downloaded at https://www.mdpi.com/article/10.3390/v18090977/s1: Table S1: Key mutation sites in H9N2 AIVs.

## References

[1] Homme P.J., Easterday B.C. (1970). Avian influenza virus infections. I. Characteristics of influenza A-turkey-Wisconsin-1966 virus. Avian Dis..

[2] Brown I.H., Banks J., Manvell R.J., Essen S.C., Shell W., Slomka M., Londt B., Alexander D.J. (2006). Recent epidemiology and ecology of influenza A viruses in avian species in Europe and the Middle East. Dev. Biol..

[3] Xiao Y., Yang F., Liu F., Yao H., Wu N., Wu H. (2021). Antigen-capture ELISA and immunochromatographic test strip to detect the H9N2 subtype avian influenza virus rapidly based on monoclonal antibodies. Virol. J..

[4] Zhang C., Xuan Y., Shan H., Yang H., Wang J., Wang K., Li G., Qiao J. (2015). Avian influenza virus H9N2 infections in farmed minks. Virol. J..

[5] El Mellouli F., Mouahid M., Fusaro A., Zecchin B., Zekhnini H., El Khantour A., Giussani E., Palumbo E., Rguibi Idrissi H., Monne I. (2022). Spatiotemporal Dynamics, Evolutionary History and Zoonotic Potential of Moroccan H9N2 Avian Influenza Viruses from 2016 to 2021. Viruses.

[6] Horm S.V., Tarantola A., Rith S., Ly S., Gambaretti J., Duong V.Y.P., Sorn S., Holl D., Allal L., Kalpravidh W. (2016). Intense circulation of A/H5N1 and other avian influenza viruses in Cambodian live-bird markets with serological evidence of sub-clinical human infections. Emerg. Microbes Infect..

[7] Carnaccini S., Perez D.R. (2020). H9 Influenza Viruses: An Emerging Challenge. Cold Spring Harb. Perspect. Med..

[8] Guan Y., Shortridge K.F., Krauss S., Webster R.G. (1999). Molecular characterization of H9N2 influenza viruses: Were they the donors of the “internal” genes of H5N1 viruses in Hong Kong?. Proc. Natl. Acad. Sci. USA.

[9] Ye G., Liang C.H., Hua D.G., Song L.Y., Xiang Y.G., Guang C., Lan C.H., Ping H.Y. (2016). Phylogenetic Analysis and Pathogenicity Assessment of Two Strains of Avian Influenza Virus Subtype H9N2 Isolated from Migratory Birds: High Homology of Internal Genes with Human H10N8 Virus. Front. Microbiol..

[10] Bhat S., James J., Sadeyen J.R., Mahmood S., Everest H.J., Chang P., Walsh S.K., Byrne A.M.P., Mollett B., Lean F. (2022). Coinfection of Chickens with H9N2 and H7N9 Avian Influenza Viruses Leads to Emergence of Reassortant H9N9 Virus with Increased Fitness for Poultry and a Zoonotic Potential. J. Virol..

[11] Yehia N., Ibrahim M., Shady R.M., Mohamed A.A.E., Said D., Taha M.E., Arafa A., Eid S., Shalaby M.A., Truyen U. (2026). Concurrent circulation of avian influenza viruses H5N1 and H9N2 enhances the genetic evolution of reassortant viruses in Egyptian poultry populations. PLoS ONE.

[12] Peacock T.H.P., James J., Sealy J.E., Iqbal M. (2019). A global perspective on H9N2 avian influenza virus. Viruses.

[13] Zhang W., Sun L., Wang Q., Xia C., Zhao Y. (2026). Molecular characterization and pathogenicity of H9N2 avian influenza viruses in poultry in Shandong Province, China, 2021–2023. Poult. Sci..

[14] Islam A., Amin E., Khan M.A., Islam M., Gupta S.D., Abedin J., Rahman M.Z., Forwood J.K., Hosaain M.E., Shirin T. (2025). Epidemiology and evolutionary dynamics of H9N2 avian influenza virus in Bangladesh. Emerg. Microbes Infect..

[15] Sun X., Belser J.A., Maines T.R. (2020). Adaptation of H9N2 Influenza Viruses to Mammalian Hosts: A Review of Molecular Markers. Viruses.

[16] Dong J., Zhou Y., Pu J., Liu L. (2022). Status and Challenges for Vaccination against Avian H9N2 Influenza Virus in China. Life.

[17] Dong G., Peng C., Luo J., Wang C., Han L., Wu B., Ji G., He H. (2015). Adamantane-resistant influenza A viruses in the world (1902–2013): Frequency and distribution of M2 gene mutations. PLoS ONE.

[18] Kumar S., Stecher G., Tamura K. (2016). MEGA7: Molecular Evolutionary Genetics Analysis Version 7.0 for Bigger Datasets. Mol. Biol. Evol..

[19] Zhang J., Su R., Jian X., An H., Jiang R., Mok C.K.P. (2018). The D253N Mutation in the Polymerase Basic 2 Gene in Avian Influenza (H9N2) Virus Contributes to the Pathogenesis of the Virus in Mammalian Hosts. Virol. Sin..

[20] Liu Q., Huang J., Chen Y., Chen H., Li Q., He L., Hao X., Liu J., Gu M., Hu J. (2015). Virulence determinants in the PB2 gene of a mouse-adapted H9N2 virus. J. Virol..

[21] Wang C., Lee H.H.Y., Yang Z.F., Mok C.K.P., Zhang Z. (2016). PB2-Q591K mutation determines the pathogenicity of avian H9N2 influenza viruses for mammalian species. PLoS ONE.

[22] Sediri H., Thiele S., Schwalm F., Gabriel G., Klenk H.D. (2016). PB2 subunit of avian influenza virus subtype H9N2: A pandemic risk factor. J. Gen. Virol..

[23] Gao W., Zu Z., Liu J., Song J., Wang X., Wang C., Liu L., Tong Q., Wang M., Sun H. (2019). Prevailing I292VPB2 mutation in avian influenza H9N2 virus increases viral polymerase function and attenuates IFN-β induction in human cells. J. Gen. Virol..

[24] Kamiki H., Matsugo H., Kobayashi T., Ishida H., Takenaka-Uema A., Murakami S., Horimoto T. (2018). A PB1-K577E mutation in H9N2 influenza virus increases polymerase activity and pathogenicity in mice. Viruses.

[25] Ma L., Zheng H., Ke X., Gui R., Yao Z., Xiong J., Chen Q. (2024). Mutual antagonism of mouse-adaptation mutations in HA and PA proteins on H9N2 virus replication. Virol. Sin..

[26] Xu G., Zhang X., Gao W., Wang C., Wang J., Sun H., Sun Y., Guo L., Zhang R., Chang K.C. (2016). Prevailing PA mutation K356R in avian influenza H9N2 virus increases mammalian replication and pathogenicity. J. Virol..

[27] Sang X., Wang A., Ding J., Kong H., Gao X., Li L., Chai T., Li Y., Zhang K., Wang C. (2015). Adaptation of H9N2 AIV in guinea pigs enables efficient transmission by direct contact and inefficient transmission by respiratory droplets. Sci. Rep..

[28] Wang C., Qu R., Zong Y., Qin C., Liu L., Gao X., Sun H., Sun Y., Chang K.C., Zhang R. (2022). Enhanced stability of M1 protein mediated by a phospho-resistant mutation promotes the replication of prevailing avian influenza virus in mammals. PLoS Pathog..

[29] Schnell J.R., Chou J.J. (2008). Structure and mechanism of the M2 proton channel of influenza A virus. Nature.

[30] Ma W., Ren C., Shi L., Meng B., Feng Y., Zhang Y. (2025). Isoleucine at position 137 of haemagglutinin acts as a mammalian adaptation marker of H9N2 avian influenza virus. Emerg. Microbes Infect..

[31] Guo Y., Bai X., Liu Z., Liang B., Zheng Y., Dankar S., Ping J. (2023). Exploring the alternative virulence determinants PB2 S155N and PA S49Y/D347G that promote mammalian adaptation of the H9N2 avian influenza virus in mice. Vet. Res..

[32] Jin F., Dong X., Wan Z., Ren D., Liu M., Geng T., Zhang J., Gao W., Shao H., Qin A. (2019). A Single Mutation N166D in Hemagglutinin Affects Antigenicity and Pathogenesis of H9N2 Avian Influenza Virus. Viruses.

[33] Teng Q., Xu D., Shen W., Liu Q., Rong G., Li X., Yan L., Yang J., Chen H., Yu H. (2016). A Single Mutation at Position 190 in Hemagglutinin Enhances Binding Affinity for Human Type Sialic Acid Receptor and Replication of H9N2 Avian Influenza Virus in Mice. J. Virol..

[34] Wan H., Perez D.R. (2007). Amino acid 226 in hemagglutinin of H9N2 influenza viruses determines cell tropism and replication in human airway epithelial cells. J. Virol..

[35] Liu Y., Zeng Q., Hu X., Xu Z., Pan C., Liu Q., Yu J., Wu S., Sun M., Liao M. (2023). Natural variant R246K in hemagglutinin increased zoonotic characteristics and renal inflammation in mice infected with H9N2 influenza virus. Vet. Microbiol..

[36] Liu Y., Li S., Sun H., Pan L., Cui X., Zhu X., Feng Y., Li M., Yu Y., Wu M. (2020). Variation and Molecular Basis for Enhancement of Receptor Binding of H9N2 Avian Influenza Viruses in China Isolates. Front. Microbiol..

[37] Zheng Y., Guo Y., Li Y., Liang B., Sun X., Li S., Xia H., Ping J. (2022). The molecular determinants of antigenic drift in a novel avian influenza A (H9N2) variant virus. Virol. J..

[38] Kode S.S., Pawar S.D., Cherian S.S., Tare D.S., Bhoye D., Keng S.S., Mullick J. (2019). Selection of avian influenza A (H9N2) virus with reduced susceptibility to neuraminidase inhibitors oseltamivir and zanamivir. Virus Res..

[39] Cao Y., Liu H., Liu D., Liu W., Luo T., Li J. (2022). Hemagglutinin Gene Variation Rate of H9N2 Avian Influenza Virus by Vaccine Intervention in China. Viruses.

[40] Yang J., Müller N.F., Bouckaert R., Xu B., Drummond A.J. (2019). Bayesian phylodynamics of avian influenza A virus H9N2 in Asia with time-dependent predictors of migration. PLoS Comput. Biol..

[41] Youk S.S., Lee D.H., Jeong J.H., Pantin-Jackwood M.J., Song C.S., Swayne D.E. (2020). Live bird markets as evolutionary epicentres of H9N2 low pathogenicity avian influenza viruses in Korea. Emerg. Microbes Infect..

[42] Fusaro A., Monne I., Salviato A., Valastro V., Schivo A., Amarin N.M., Gonzalez C., Ismail M.M., Al-Ankari A.R., Al-Blowi M.H. (2011). Phylogeography and evolutionary history of reassortant H9N2 viruses with potential human health implications. J. Virol..

[43] Machalaba C.C., Elwood S.E., Forcella S., Smith K.M., Hamilton K., Jebara K.B., Swayne D.E., Webby R.J., Mumford E., Mazet J.A. (2015). Global avian influenza surveillance in wild birds: A strategy to capture viral diversity. Emerg. Infect. Dis..

[44] Hoye B.J., Munster V.J., Nishiura H., Klaassen M., Fouchier R.A. (2010). Surveillance of wild birds for avian influenza virus. Emerg. Infect. Dis..

[45] Murakami J., Shibata A., Neumann G., Imai M., Watanabe T., Kawaoka Y. (2022). Characterization of H9N2 Avian Influenza Viruses Isolated from Poultry Products in a Mouse Model. Viruses.

[46] Kale S.D., Mishra A.C., Pawar S.D. (2013). Suitability of specimen types for isolation of avian influenza viruses from poultry. Indian J. Virol..

[47] Qi Y., Guo W., Liu C., Li W., Gu Y., Li S., Chen X. (2021). Seroprevalence of influenza A (H9N2) virus infection among humans in China: A meta-analysis. Microb. Pathog..

[48] Wang Q., Xu K., Xie W., Yang L., Chen H., Shi N., Bao C., Huang H., Zhang X., Liao Y. (2020). Seroprevalence of H7N9 infection among humans: A systematic review and meta-analysis. Influenza Other Respir. Viruses.

[49] Sun W., Cheng S.S.M., Lam K.N.T., Kwan T.C., Wong R.W.K., Lau L.H.K., Liu G.Y.Z., Luk L.L.H., Li J.K.C., Gu H. (2022). Natural Reassortment of Eurasian Avian-Like Swine H1N1 and Avian H9N2 Influenza Viruses in Pigs, China. Emerg. Infect. Dis..

[50] Peiris M., Yuen K.Y., Leung C.W., Chan K.H., Ip P.L., Lai R.W., Orr W.K., Shortridge K.F. (1999). Human infection with influenza H9N2. Lancet.

[51] Sun Y., Pu J., Fan L., Sun H., Wang J., Zhang Y., Liu L., Liu J. (2012). Evaluation of the protective efficacy of a commercial vaccine against different antigenic groups of H9N2 influenza viruses in chickens. Vet. Microbiol..

[52] Hussein A.F.A., Cheng H., Tundup S., Antanasijevic A., Varhegyi E., Perez J., AbdulRahman E.M., Elenany M.G., Helal S., Caffrey M. (2020). Identification of entry inhibitors with 4-aminopiperidine scaffold targeting group 1 influenza A virus. Antivir. Res..

[53] Yang J., Xie D., Nie Z., Xu B., Drummond A.J. (2019). Inferring host roles in Bayesian phylodynamics of global avian influenza A virus H9N2. Virology.

