# Supplementary material for: Global Patterns of Geographic Distribution, Temporal Trends, Host Spectrum, and Molecular Variation of H9N2 Avian Influenza Virus, 1966–2023
Source: Viruses. 2026 Sep 4;18(9):977. doi: 10.3390/v18090977 (PMC13612056; doi:10.3390/v18090977)
Supplement: Supplementary file 1 [file viruses-18-00977-s001.zip › viruses-4446640-supplementary.pdf]

Table S1. Key mutation sites in H9N2 AIVs.

Protein	Reported mutation sites	Corresponding amino acid distribution in database
**PB2**	D253N	D (4417); E (11); N (5)
	I292V	A (13); E (1); G (2); I (1733); L (1); M (13); T (149); V (2509)
	F404L	F (4738); L (1)
	Q591K	Q (4661); L (102); K (51); P (5); H (1); R (2)
	E627K, D701N, S714R	627: A (2); E (4503); G (1); K (26); V (287); 701: D (4753); E (11); N (16); V (1); 714: S (4610); G (141); T (20); N (11); C (2)
**PB1**	K577E	K (4411); R (23); Q (2); E (3); N (6)
**PA**	T97I, I545V, S594G	97: T (4355); A (92); N (28); I (3); V (3); K (1); S (1); 545: I (4532); V (267); K (1); 594: S (4760); G (5); N (5); R (2)
	K356R	K (2720); R (2142); E (1); T (1)
**NP**	E434K	E (4554); K (1); G (2); A (1); D (8)
**M1**	T37A	T (1677); A (3173); P (2); I (8); V (166); S (1)
**M2**	S31N	S (1688); N (3355); G (5); I (3); R (3); D (3); C (2)
**HA**	T137I	Y (2802); K (1); H (1); S (5132); T (1495); D (4321); N (371); G (13); P (1); I (1); R (7); L (1); A (6); C (6); E (8)
	I155T	T (11592); A (5); S (8); F (2); I (7); Y (2); N (50)
	N166D	A (1); T (7); V (1); Y (1); D (2120); E (3); G (46); H (1); K (1); N (8658); Q (1); R (20); S (799)
	A190V	S (9); L (7); N (4); Q (6); A (4644); V (1178); T (5110); E (651); G (11); D (31); M (2); I (8); K (39)
	Q226L	Q (1108); L (10562); M (14); H (4); P (2); F (4); S (3); R (4); V (1)
	R246K	R (5279); K (6408); Q (3); M (4); I (16); S (3); G (1); E (2); N (6); T (1); Y (1)
	Q227M、D145G/N、S119R、R246K；A160D/N、Q156R、T205A、Q226L、V245I、V216L、D208E、T212I、R172Q、S175N	227: A (6); E (1); F (4); H (7); I (1802); K (1); L (8); M (6710); N (1); Q (3099); R (16); T (50); V (1); 145: G (4352); D (6116); N (1056); Q (2); S (88); V (3); E (12); H (2); R (12); Y (2); 119: S (3891); R (7241); K (95); N (323); T (21); G (70); C (1); P (1); M (5); I (3); H (1); E (2); Q (1); 246: R (5279); K (6408); Q (3); M (4); I (16); S (3); G (1); E (2); N (6); T (1);Y (1); 160: T (147); A (4109); G (88); N (3005); S (704); F (154); D (1312); L (735); V (92); Q (22); E (1261); K (5); I (8); H (7); 156: Q (6756); R (4256); H (566); E (4); K (41); P (24); L (15); 205: A (8853); T (2827); S (13); V (11); M (4); K (1); G (3); E (1); R (1); 226: Q (1108); L (10562); M (14); H (4); P (2); F (4); S (3); R (4); V (1); 245: V (3350); I (8362); L (6); M (2); G (1); K (1); N (1); 216: A (12); L (10569); V (915); M (165); S (29); E (1); I (5); Y (1); F (3); G (1); 208: D (1855); T (152); E (8082); Y (6); N (1242); S (372); I (1); G (3); K (2); 212: T (5388); I (5665); A (93); V (536); S (10); N (10); G (1); 172: R (3137); Q (7857); E (309); K (43); V (3); W (213); G (34); I (3); M (7); L (15); N (2); H (9); S (1); P (19); 175: D (1854); N (9099); S (681); H (6); R (1); G (5); T (5); I (2); K (3)
	D127S、G135D、N145T、R146Q、D179T、R182T、T183N	127: S (5132); T (1495); D (4321); N (371); G (13); P (1); A (6); L (1); I (1); R (7); E (8); Y (280); C (6); K (1); H (1); 135: D (6116); N (1056); Q (2); G (4352); S (88); V (3); E (12); H (2); R (12); Y (2); 145: A (5); T (11592); F (2); I (7); S (8); Y (2); N (50); 146: Q (6756); R (4256); H (566); K (41); E (4); P (24); L (15); 179: T (8709); A (91); D (2454); N (140); S (28); P (3); I (6); G (5); Y (5); V (1); E (278); K (5); 182: T (6938); R (4060); I (160); N (38); K (13); M (199); A (9); S (262); G (7); E (33); 183: N (8314); D (1320); S (492); E (204); T (114); A (51); Q (1); G (1144); K (36); H (1); V (1); R (23); I (6)
**NA**	E119D; R292K	119: D (3); E (5805); V (1); K (5); Q (1); 292: R (5854); I (2); W (5); G (1); T (2); L (1)
